# *ZmbHLH81* Enhances Maize Drought Tolerance via Direct Transcriptional Activation of ABA Signaling and ROS Scavenging Genes

**DOI:** 10.3390/ijms27073293

**Published:** 2026-04-05

**Authors:** Nannan Zhang, Guanfeng Wang, Xinping Zhang, Wenzhe Zhao, Qi Shi, Xiaowei Fan, Nan Lin, Song Song

**Affiliations:** 1 College of Life Sciences, Henan Agricultural University, Zhengzhou 450002, China; 2 College of Agronomy and Biotechnology, China Agricultural University, Beijing 100193, China; 3 Sanya Institute of China Agricultural University, Sanya 572024, China

**Keywords:** maize (*Zea mays* L.), drought, ZmbHLH81, transcription factor, ABA, stomatal closure, ROS

## Abstract

Drought severely limits maize production. Basic helix-loop-helix (bHLH) transcription factors act as key regulators of plant drought responses; however, the precise regulatory networks they coordinate in maize remain largely unclear. Here, we functionally characterized ZmbHLH81, a drought- and abscisic acid (ABA)-responsive bHLH transcription factor in maize. Subcellular localization confirmed that ZmbHLH81 is a nuclear protein. Overexpression of *ZmbHLH81* in Arabidopsis enhanced drought tolerance, whereas CRISPR/Cas9-mediated targeted mutagenesis in maize significantly increased plant sensitivity to drought stress. Physiologically, these mutant lines exhibited accelerated water loss, delayed stomatal closure, compromised antioxidant enzyme activities and elevated malondialdehyde (MDA) accumulation under drought stress. DAP-seq analysis demonstrated that ZmbHLH81 specifically recognizes the conserved G-box motif (CACGTG). Furthermore, integrating DAP-seq and transcriptomic data successfully identified the key downstream targets governed by ZmbHLH81. Molecular assays confirmed that ZmbHLH81 directly targets and transactivates the core ABA signaling kinase gene *ZmSnRK2.9* and stress-responsive transcription factor genes *ZmNAC20* and *ZmHDZ4*. Taken together, ZmbHLH81 positively regulates maize drought tolerance by directly activating a specific regulatory module that orchestrates ABA-mediated stomatal closure and reactive oxygen species (ROS) scavenging, providing a promising genetic target for breeding climate-resilient crops.

## 1. Introduction

Drought is a major environmental factor that limits crop growth and agricultural productivity [1,2]. Maize (*Zea mays* L.) is an important food and feed crop worldwide, but its production is highly sensitive to water deficit [3]. Severe drought stress disrupts normal physiological processes in plants, ultimately leading to significant yield losses [4,5]. However, the number of characterized genes available for maize drought-resistance breeding remains limited. Therefore, identifying drought-responsive genes and understanding the underlying molecular mechanisms are essential for the genetic improvement of drought tolerance in maize [6,7].

To survive under water-limiting conditions, plants have evolved physiological and biochemical defense mechanisms. The phytohormone abscisic acid (ABA) acts as a central regulator in this stress adaptation process [8]. Under drought stress, endogenous ABA levels increase rapidly, initiating a core signal transduction pathway [9]. In this pathway, ABA is perceived by Pyrabactin Resistance 1/PYR1-Like (PYR/PYL) receptors, which then bind to and inactivate clade A protein phosphatases type 2C (PP2Cs) [10]. This interaction relieves the inhibition of sucrose non-fermenting 1 (SNF1)-related protein kinases type 2 (SnRK2s) [11]. The activated SnRK2 kinases subsequently phosphorylate distinct downstream targets: they modulate ion channels to trigger rapid stomatal closure, and activate transcription factors to induce stress-responsive gene expression [12,13]. Alongside water deficit, drought stress typically disrupts cellular redox balance, causing the overproduction of reactive oxygen species (ROS) [14]. Excessive ROS accumulation results in oxidative damage to cell membranes, proteins, and DNA [15]. To prevent this cellular toxicity, plants activate an antioxidant defense system. This defense mechanism involves the upregulation of ROS-scavenging enzymes, such as superoxide dismutase (SOD) and peroxidase (POD), to clear excess ROS and maintain cellular redox homeostasis [16,17,18]. Therefore, the ability to regulate ABA-mediated stomatal closure and clear excessive ROS largely determines plant drought tolerance [14].

In addition to these physiological and biochemical defenses, plant drought adaptation relies on extensive transcriptional reprogramming [19]. Transcription factors serve as essential regulators in this process by binding to specific *cis*-elements to modulate the expression of downstream stress-responsive genes [20]. To date, members from diverse transcription factor families, such as NAM, ATAF1/2, and CUC2 (NAC), basic leucine zipper (bZIP), APETALA2/ethylene-responsive factor (AP2/ERF), myeloblastosis (MYB), WRKY, and others, have been widely identified as key components in plant drought responses [9,21,22,23,24,25,26]. The basic helix-loop-helix (bHLH) proteins constitute one of the largest transcription factor families in eukaryotes, characterized by a highly conserved basic region for DNA binding and an HLH domain for protein dimerization [27,28]. They typically regulate downstream targets by specifically recognizing the E-box (CANNTG) or G-box (CACGTG) motifs in promoter regions [29]. While bHLH proteins are well known for regulating plant growth and development, they also play important roles in plant responses to drought stress [30]. In plants, these transcription factors frequently coordinate phytohormone pathways, particularly ABA signaling, to dictate specific stress responses like stomatal movement and antioxidant defense [31]. By forming homo- or heterodimers, bHLH proteins often assemble into multi-tiered regulatory cascades, allowing plants to finely tune the trade-off between normal growth and stress survival [28,32]. For instance, specific members such as *OsbHLH148* in rice, *TabHLH27* in wheat, and *ZmPTF1* in maize have been proven to positively regulate drought tolerance [33,34,35]. However, despite the large number of predicted bHLH members in the maize genome, only a small fraction has been functionally characterized under abiotic stress conditions.

Here, we functionally characterized the drought- and ABA-responsive maize transcription factor ZmbHLH81. Analyses of CRISPR/Cas9-generated mutants and overexpression lines demonstrated that *ZmbHLH81* positively regulates drought tolerance by mitigating water loss and oxidative damage. By integrating DAP-seq and RNA-seq with molecular assays, we identified key downstream targets of ZmbHLH81. Specifically, we found that ZmbHLH81 directly transactivates the core ABA signaling kinase gene *ZmSnRK2.9* and the stress-responsive transcription factor genes *ZmNAC20* and *ZmHDZ4*. The activation of these specific targets functions to promote rapid stomatal closure and reactive oxygen species (ROS) scavenging. These findings clarify the molecular mechanism of *ZmbHLH81* and provide a precise genetic target for breeding climate-resilient maize.

## 2. Results

### 2.1. Expression Pattern and Subcellular Localization of ZmbHLH81

**Figure 1:**
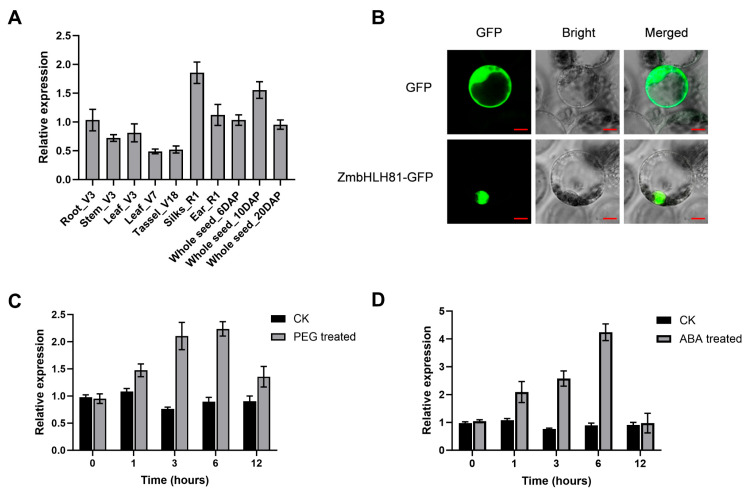
RNA expression pattern and subcellular localization of ZmbHLH81. (**A**) Relative expression levels of *ZmbHLH81* in various maize tissues at different developmental stages. V3, V7, V18, and R1 indicate the vegetative 3, vegetative 7, vegetative 18, and reproductive 1 stage, respectively. DAP represents days after pollination. (**B**) Subcellular localization of the ZmbHLH81 fusion protein in maize leaf protoplasts. The ZmbHLH81-GFP fusion protein and the empty GFP control were transiently expressed in maize leaf protoplasts. The green fluorescent signal of ZmbHLH81-GFP was exclusively localized to the nucleus. Scale bars: 10 μm. (**C**,**D**) Expression analysis of *ZmbHLH81* in maize seedling leaves subjected to 10% PEG6000 (**C**) and 100 μM ABA (**D**) treatments. Error bars represent the standard deviation (SD) based on three biological replicates.

To gather evidence on the putative biological function of *ZmbHLH81* in maize, we first characterized its expression profile in multiple tissues at different developmental stages. This showed that *ZmbHLH81* was expressed in all examined tissues, including roots, stems, and leaves at the V3 stage, leaves at the V7 stage, tassels at the V18 stage, silks and ears at the R1 stage, and whole developing seeds at 6, 10, and 20 days after pollination (DAP), without obvious tissue specificity (Figure 1A). To determine the subcellular localization of ZmbHLH81, a ZmbHLH81-green fluorescent protein (GFP) fusion construct was generated and introduced into maize leaf protoplasts isolated from 10-day-old etiolated maize seedlings. As shown in Figure 1B, the green fluorescent signal of ZmbHLH81-GFP was exclusively detected in the nucleus. This result indicated that ZmbHLH81 is a nuclear protein, which is consistent with its putative functional role as a bHLH transcription factor.

Next, we examined the expression levels of *ZmbHLH81* under osmotic stress and ABA treatments to investigate its potential involvement in these stress responses. The results showed that the transcript level of *ZmbHLH81* in the leaves of seedlings at the three-leaf stage was significantly upregulated after treatment with 10% PEG6000 (Figure 1C). Furthermore, exogenous application of 100 µM ABA also rapidly induced the expression of *ZmbHLH81* (Figure 1D). This rapid induction followed by a decline at 12 h represents a typical transient expression profile characteristic of early stress-responsive transcription factors, reflecting a rapid initial activation of downstream targets followed by natural signal attenuation to maintain cellular homeostasis. Together, these expression analyses indicated that *ZmbHLH81* is an osmotic stress- and ABA-responsive gene, suggesting its potential role in maize drought tolerance.

### 2.2. ZmbHLH81 Positively Regulates Drought Tolerance in Arabidopsis Overexpression Lines

**Figure 2:**
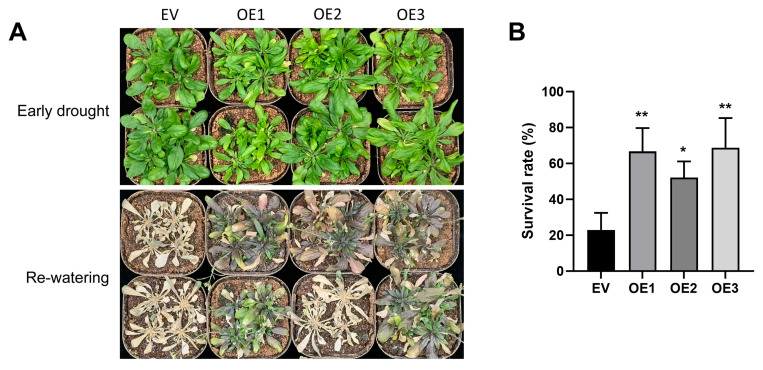
Overexpression of *ZmbHLH81* improves drought tolerance in *Arabidopsis* transformant lines. (**A**) Phenotypes of the empty vector (EV) control and *ZmbHLH81*-overexpressing (OE) lines at the early stage of drought and after re-watering. (**B**) Survival rates of the EV and OE lines after drought treatment. Values represent the means ± SD of three independent biological replicates. *, *p* < 0.05, **, *p* < 0.01, as determined by one-way ANOVA followed by Fisher’s LSD test.

To evaluate whether *ZmbHLH81* confers drought tolerance, we generated *Arabidopsis thaliana* (*Arabidopsis*) transformant lines overexpressing *ZmbHLH81* (OE). 20-day-old plants of three independent homozygous OE lines and an empty vector (EV) control were subjected to drought stress by withholding water for 9 days. After re-watering for 7 days, the survival rates were recorded. This showed that the survival rates of the three OE lines (averaging over 50%) were significantly higher than that of the EV control (~20%) (Figure 2A,B), a finding which readily demonstrated that overexpression of *ZmbHLH81* in *Arabidopsis* enhanced its tolerance to drought stress.

**Figure 3:**
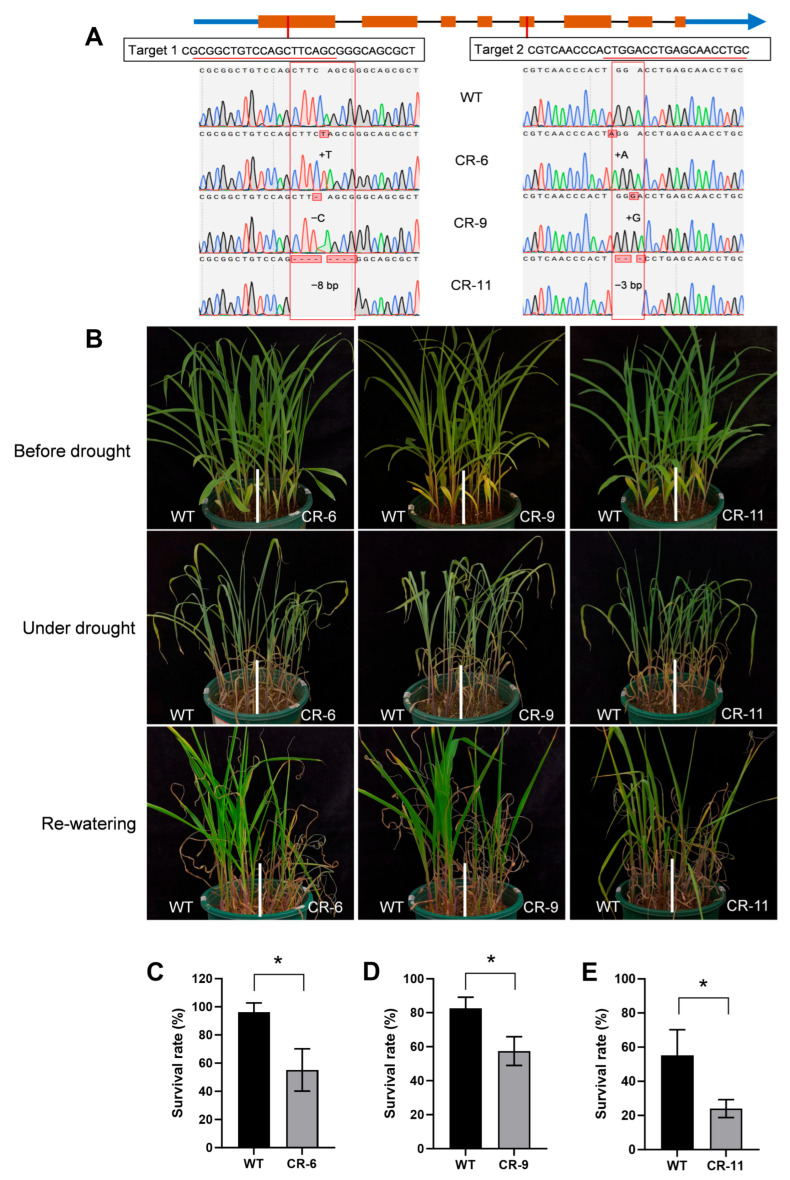
CRISPR/Cas9-mediated mutation of *ZmbHLH81* decreases drought tolerance in maize. (**A**) Schematic diagram of the CRISPR/Cas9 target sites and the specific mutations in the three independent homozygous CR lines (CR-6, CR-9, and CR-11). In the gene structure model, orange boxes represent exons, the black line represents introns, and blue lines/arrows indicate untranslated regions (UTRs). The red boxes highlight the inserted or deleted nucleotides. (**B**) Phenotypes of the WT and CR lines before drought, under drought stress, and after re-watering. (**C**–**E**) Survival rates of the CR-6 (**C**), CR-9 (**D**), and CR-11 (**E**) lines compared with the WT after re-watering. Error bars represent the SD based on three biological replicates. *, *p* < 0.05, Student’s *t*-test.

To further investigate the biological role of *ZmbHLH81* in maize, we generated targeted mutants (hereafter referred to as CR lines) in the B104 inbred line using the CRISPR/Cas9 system. Two target sites located in the first and fifth exons were designed. We obtained three independent homozygous mutant lines, designated as CR-6, CR-9, and CR-11. Sanger sequencing confirmed that the simultaneous indels at both target sites caused frameshift mutations, leading to either premature translation termination (in CR-6 and CR-9) or a completely altered amino acid sequence (in CR-11), both of which are predicted to disrupt the function of the ZmbHLH81 protein (Figure 3A and Figure S1). The three CR lines and wild-type (WT) plants were subjected to drought stress at the three-leaf stage. During the stress period, all CR lines exhibited more severe wilting and leaf rolling phenotypes than the WT, and the survival rates of the three CR lines were significantly lower than that of the WT at 5 days after re-watering (Figure 3B–E). Taken together, these loss-of-function results further confirmed that *ZmbHLH81* acts as a positive regulator of drought tolerance in maize, and its absence significantly increases plant sensitivity to water deficit.

### 2.3. ZmbHLH81 Promotes Stomatal Closure and Maintains Antioxidant Enzyme Activities Under Drought

**Figure 4:**
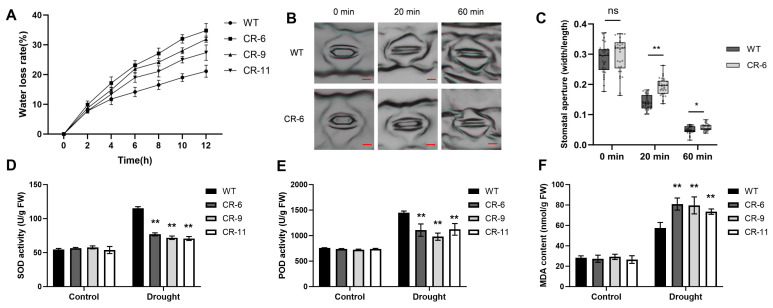
Physiological responses of the WT and the mutant lines under drought stress. (**A**) Water loss rates of detached leaves from the WT and three mutant lines (CR-6, CR-9, and CR-11). The second fully expanded leaves at the three-leaf stage were sampled for the assay. The leaf weight was recorded every 2 h during a 12-h dehydration period. (**B**,**C**) Stomatal closure assays. Scale bars: 10 μm. Representative images of stomata (**B**) and statistical analysis of the stomatal aperture (width/length ratio) (**C**) in the WT and CR-6 after dehydration for 0, 20, and 60 min. The detached leaves were pre-incubated in a stomatal opening buffer for 3 h in the dark and then subjected to dehydration under light. (**D**–**F**) Activities of SOD (**D**), POD (**E**), and MDA content (**F**) in the leaves of the WT and three mutant lines under well-watered (Control) and drought conditions. Values represent the means ± SD based on five biological replicates. Statistical significance was determined using Student’s *t*-test for (**C**), and two-way ANOVA followed by Fisher’s LSD test for (**D**–**F**) (ns, not significant; * *p* < 0.05; ** *p* < 0.01 vs. WT).

Plant drought tolerance closely relies on the capacity to maintain water balance and prevent cellular damage. To investigate the physiological basis of the drought-sensitive phenotype in the CRISPR/Cas9-mediated mutant lines, we initially measured the water loss rate of detached leaves at the three-leaf stage. As shown in Figure 4A, the detached leaves of all three CR lines exhibited a consistently higher water loss rate than those of the WT during a 12-h dehydration period. This result indicated that the mutant lines had a reduced water retention capacity.

Water loss in leaves is primarily controlled by changes in stomatal aperture. Therefore, we examined the stomatal aperture during dehydration. Given that all three independent mutant lines exhibited consistent drought-sensitive phenotypes at the whole-plant level, CR-6 was utilized as a representative line for subsequent physiological assays. The detached leaves of the WT and the mutant were first incubated in a stomatal opening buffer in the dark, followed by dehydration under light. At 0 min, the stomatal apertures of the WT and the mutant were comparable. However, after dehydration for 20 and 60 min, the stomata of the CR-6 line closed much slower, resulting in significantly larger stomatal apertures compared with the WT (Figure 4B,C). The delayed stomatal closure suggested that *ZmbHLH81* promotes stomatal closure to minimize water loss during dehydration.

Drought stress typically triggers the accumulation of ROS, and plants rely on antioxidant enzymes, such as SOD and POD, to alleviate oxidative damage. We measured the SOD and POD activities in the leaves of three-leaf stage WT and the three CR lines. Under well-watered conditions, no significant difference in SOD and POD activities was observed among the genotypes. Following drought treatment, although the activities of both enzymes were induced, the SOD and POD activities in all three CR lines were significantly lower than those determined for WT plants (Figure 4D,E). To further evaluate the resulting cell membrane damage, we also measured the malondialdehyde (MDA) content. Similarly to the enzyme activities, MDA levels showed no significant difference under normal conditions but were significantly higher in all three CR lines compared to the WT after drought stress (Figure 4F). These results demonstrated that the loss of *ZmbHLH81* function compromised the antioxidant enzyme system and exacerbated lipid peroxidation, rendering the plants more susceptible to drought-induced oxidative stress.

### 2.4. Transcriptome Profiling of the ZmbHLH81-Mediated Drought Response Network

**Figure 5:**
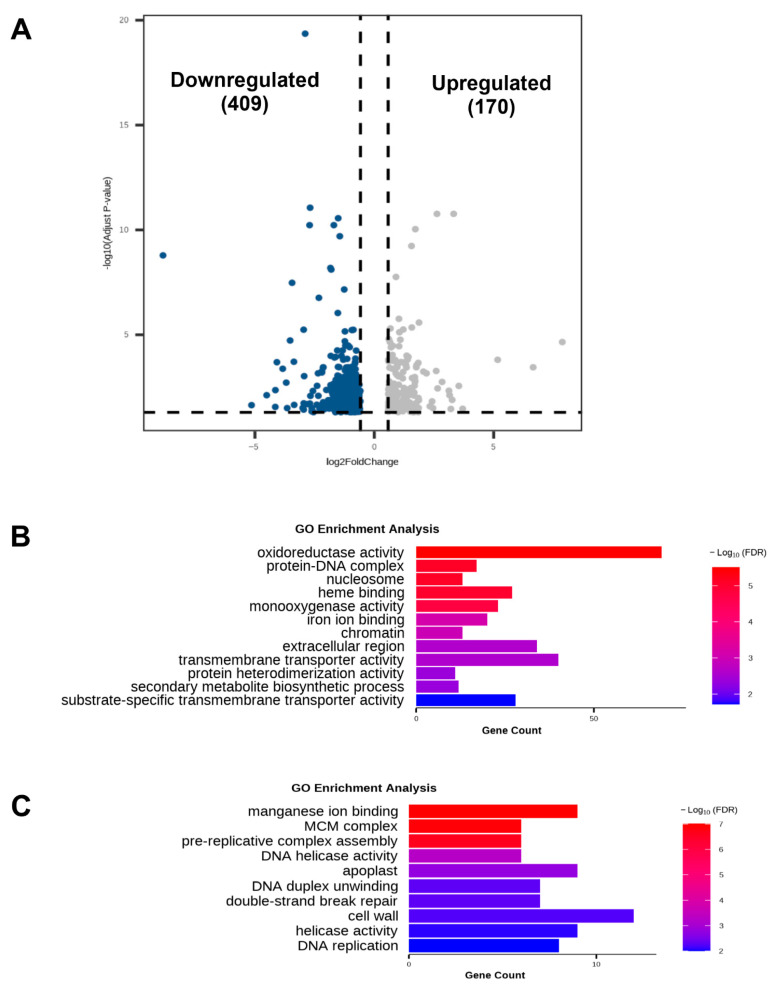
Transcriptome profiling of the WT and *ZmbHLH81* CRISPR mutant under drought stress. (**A**) Volcano plot showing the DEGs in the CR-6 mutant compared with the WT. The blue and gray dots indicate significantly downregulated (409) and upregulated (170) genes in the mutant, respectively. The dashed lines represent the threshold criteria for DEGs (fold change > 1.5 or <0.67, and adjusted *p* < 0.05). (**B**,**C**) Gene Ontology (GO) enrichment analysis of the 409 downregulated genes (**B**) and 170 upregulated genes (**C**) in the CR-6 mutant. The x-axis indicates the number of genes enriched in each GO category, and the color gradient represents the statistical significance level (−log_10_FDR).

To elucidate the regulatory network of *ZmbHLH81* under drought stress, we performed RNA-seq analysis using the WT and the representative mutant line (CR-6). The transcriptomic comparison identified 579 differentially expressed genes (DEGs) affected by ZmbHLH81. Compared with the WT, 409 genes were downregulated and 170 genes were upregulated in the mutant (Figure 5A; Supplementary Table S1). The number of positively regulated genes (downregulated in the CR-6 mutant line) was significantly higher than that of negatively regulated genes, indicating that *ZmbHLH81* positively affects the expression of a broad range of downstream drought-responsive genes.

We subsequently performed Gene Ontology (GO) analysis on the 409 positively regulated genes to further understand the biological functions of these DEGs. As shown in Figure 5B, the highly enriched terms were closely associated with the observed physiological phenotypes. A large proportion of these genes were enriched in antioxidant-related categories, including “oxidoreductase activity”, “heme binding” and “iron ion binding”. Furthermore, another major cluster was enriched in “transmembrane transporter activity” and “extracellular region”. Specifically, the downregulation of genes related to oxidoreductase activity and iron/heme binding likely impairs the function of antioxidant enzymes such as SOD and POD. Meanwhile, the downregulation of transmembrane transporters could restrict the ion and water efflux required for stomatal closure. Moreover, terms such as “chromatin”, “nucleosome”, and “protein heterodimerization activity” were also enriched, suggesting that ZmbHLH81 triggered a broader downstream transcriptional cascade.

In contrast, GO analysis of the 170 negatively regulated genes (upregulated in the mutant) showed a distinct functional profile (Figure 5C). These genes were primarily enriched in cell division and DNA repair processes, such as “pre-replicative complex assembly”, “DNA replication” and “double-strand break repair”. Taken together, these results suggested that the mutant failed to properly arrest energy-consuming growth processes under severe drought stress, which might exacerbate cellular damage and result in a hypersensitive phenotype.

### 2.5. Transcriptional Activity and Genome-Wide Binding Profiling of ZmbHLH81

**Figure 6:**
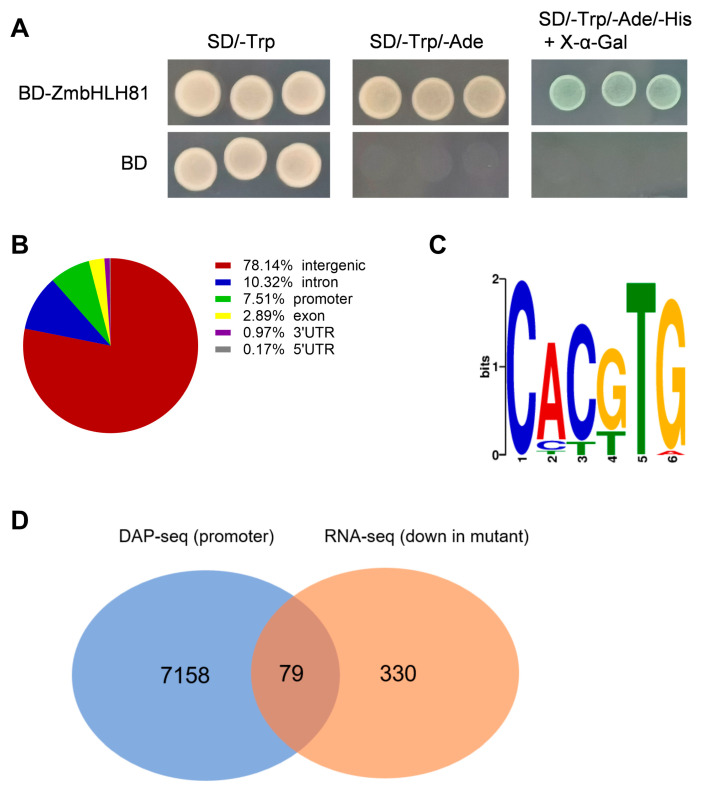
Transcriptional activity and genome-wide binding profiling of ZmbHLH81. (**A**) Transcriptional activation assay of ZmbHLH81 in yeast. Yeast cells harboring the BD-ZmbHLH81 construct or the empty BD vector (negative control) were spotted onto SD/-Trp, SD/-Trp/-Ade, and SD/-Trp/-Ade/-His (+X-α-Gal) media, respectively. (**B**) Genomic distribution of ZmbHLH81 binding peaks identified by DAP-seq. (**C**) The most highly enriched binding motif (CACGTG, typical G-box) identified from the DAP-seq peaks. (**D**) Venn diagram showing the overlap between genes harboring DAP-seq peaks in their promoter regions (7237 genes) and genes downregulated in the mutant line (409 genes, Down in mutant), identifying 79 high-confidence candidate target genes.

To investigate the transcriptional activity of ZmbHLH81, we performed a yeast transactivation assay. The full-length CDS of ZmbHLH81 was fused to the GAL4 DNA-binding domain in the pGBKT7 vector. The results showed that while all transformed yeast cells grew well on the SD/-Trp medium, only the cells expressing BD-ZmbHLH81 could survive on the selective media (SD/-Trp/-Ade and SD/-Trp/-Ade/-His) and turn blue in the presence of X-α-Gal (Figure 6A). In contrast, cells containing the empty BD vector failed to grow on the selective media. This result indicated that ZmbHLH81 possesses transcriptional activation activity in yeast.

We then performed DNA affinity purification sequencing (DAP-seq) to map the genome-wide binding sites of ZmbHLH81. Distribution analysis of the DAP-seq peaks revealed that the majority of the binding sites were located in intergenic regions, followed by introns, promoters, exons, and untranslated regions (UTRs) (Figure 6B). Motif enrichment analysis of all binding sequences identified a highly conserved sequence, CACGTG (Figure 6C). This motif is a typical G-box element, which is commonly recognized by bHLH transcription factors.

To identify candidate targets whose expression is potentially promoted by *ZmbHLH81*, we integrated the DAP-seq and RNA-seq datasets. By overlapping the 7237 genes harboring DAP-seq peaks in their promoter regions with the 409 genes downregulated in the mutant, we identified 79 candidate targets (Figure 6D). These intersecting genes represent a key subset of the regulatory network potentially mediated by *ZmbHLH81* during the drought response.

### 2.6. ZmbHLH81 Directly Targets and Activates ZmSnRK2.9, ZmNAC20, and ZmHDZ4

**Figure 7:**
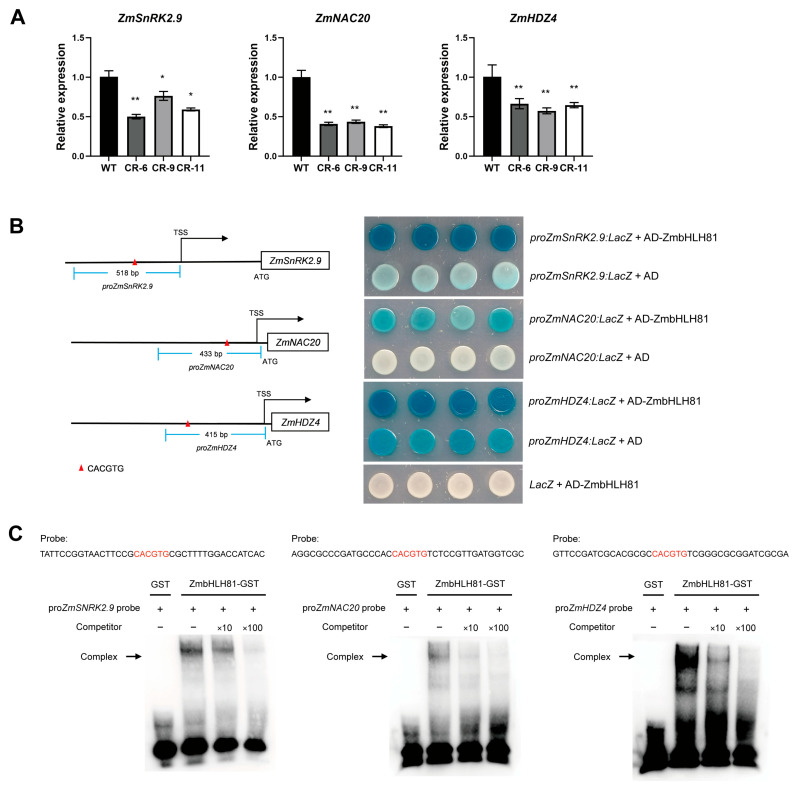
ZmbHLH81 directly binds to the promoters of *ZmSnRK2.9*, *ZmNAC20*, and *ZmHDZ4* and activates their expression. (**A**) Relative expression levels of *ZmSnRK2.9*, *ZmNAC20*, and *ZmHDZ4* in the WT and the three CR lines. Values represent the means ± SD of three biological replicates. *, *p* < 0.05; **, *p* < 0.01, as determined by one-way ANOVA followed by Fisher’s LSD test. (**B**) Y1H assays showing the binding of ZmbHLH81 to the promoters of the target genes. The schematic diagrams on the left illustrate the specific promoter fragments (blue lines, with lengths indicated) used for the Y1H assays. The red triangles indicate the positions of the G-box motifs (CACGTG) within these fragments. TSS and ATG represent the transcription start sites and translation start sites, respectively. Yeast cells co-transformed with the AD-ZmbHLH81 effector and the respective LacZ reporter constructs were grown on selective medium to detect β-galactosidase activity (blue colonies). Empty vectors (AD and LacZ) were used as negative controls. (**C**) Electrophoretic mobility shift assays (EMSAs) confirming the in vitro binding of the ZmbHLH81 protein to the G-box elements. The red letters in the probe sequences indicate the core G-box motifs. The purified ZmbHLH81-GST fusion protein was incubated with biotin-labeled probes. Unlabeled probes were used as competitors at 10× and 100× concentrations. The GST protein alone served as a negative control. Arrows indicate the formed DNA-protein complexes.

Among the candidate targets, we selected three well-characterized genes for further validation: the core ABA signaling kinase gene *SNF1-related protein kinase 2.9* (*ZmSnRK2.9*), and two transcription factor gene known to positively regulate maize drought tolerance, *NAM*, *ATAF*, and *CUC 20* (*ZmNAC20*) and *homeodomain-leucine zipper 4* (*ZmHDZ4*). qRT-PCR analysis revealed that the relative expression levels of all three genes were significantly decreased in the three CR lines compared with the WT (Figure 7A), indicating that *ZmbHLH81* positively regulates their transcription.

Sequence analysis identified the highly conserved G-box motif (CACGTG) within the promoter regions of *ZmSnRK2.9*, *ZmNAC20*, and *ZmHDZ4* (Figure 7B). To examine the direct binding, yeast one-hybrid (Y1H) assays were performed. Yeast cells co-transformed with the AD-ZmbHLH81 effector and the respective promoter-reporter constructs exhibited significantly enhanced β-galactosidase activity, resulting in visibly darker blue colonies compared with the negative controls (Figure 7B). Furthermore, the AD-ZmbHLH81 effector failed to activate the empty *LacZ* reporter, excluding the possibility of auto-activation. These results demonstrated that ZmbHLH81 recognizes these promoters and strongly activates their transcription in yeast.

To further confirm the specific binding in vitro, electrophoretic mobility shift assays (EMSA) were conducted. The purified ZmbHLH81-GST fusion protein successfully bound to the biotin-labeled probes containing the specific G-box motifs from the three promoters, forming distinct DNA-protein complexes (Figure 7C). These binding signals were competitively reduced by the addition of 10× and 100× unlabeled competitor probes in a dose-dependent manner, whereas the GST control protein showed no binding activity.

Collectively, these molecular validations corroborate our RNA-seq, DAP-seq and physiological findings. By directly targeting key genes such as *ZmSnRK2.9*, *ZmNAC20*, and *ZmHDZ4*, ZmbHLH81 participates in a downstream regulatory network to facilitate ABA signaling and drought-responsive physiological adaptations in maize.

## 3. Discussion

### 3.1. ZmbHLH81 Functions as a Positive Regulator of Drought Tolerance in Maize

bHLH transcription factors represent a major family of gene expression regulators with numerous family members associated with plant adaptation to abiotic stresses [28]. In maize, several bHLH members have been functionally characterized as regulators of drought tolerance through diverse physiological pathways. For instance, ZmbHLH124 directly binds to the promoter of *ZmDREB2A* to enhance its expression and improve drought tolerance [36]. *ZmbHLH137* positively regulates drought responses by increasing antioxidant enzyme activities, such as POD and SOD [37]. Additionally, factors like *Phytochrome-Interacting Factor 3* (*ZmPIF3*) and *ZmbHLH47* modulate water loss by participating in the ABA signaling pathway and promoting stomatal closure [38,39]. Furthermore, maize *Phosphate Transcription Factor 1* (*ZmPTF1*) acts as a positive regulator by promoting ABA synthesis and stress-responsive gene expression [33].

Consistent with the established roles of these family members, our evaluations demonstrate that *ZmbHLH81* acts as a positive regulator of drought tolerance. Physiological assessments showed that the targeted mutation of *ZmbHLH81* significantly increased the sensitivity of maize seedlings to water deficit, leading to a reduced survival rate. Conversely, overexpression of *ZmbHLH81* in *Arabidopsis* conferred enhanced drought survival. Collectively, these phenotypic results confirm the positive regulatory role of *ZmbHLH81* in the drought response.

### 3.2. ZmbHLH81 Contributes to Stomatal Regulation via the Activation of ZmSnRK2.9 and ZmNAC20

Rapid stomatal closure is a fundamental physiological response for restricting water loss under drought stress [4,12]. In this study, the *ZmbHLH81* mutants exhibited consistently higher water loss rates and delayed stomatal closure during dehydration. Consistent with these physiological observations, our molecular assays identified *ZmSnRK2.9* and *ZmNAC20* as direct downstream targets of ZmbHLH81.

The SnRK2 family members are key components of the ABA signaling pathway, primarily acting as signal transducers that modulate guard cell ion channels to facilitate stomatal movement [40]. Specifically, *ZmSnRK2.9* has been shown to actively transmit ABA signals and function as a positive regulator in maize drought tolerance [39]. Additionally, the transcription factor *ZmNAC20* has been previously characterized to improve drought resistance by specifically promoting stomatal closure and activating downstream stress-responsive genes [41]. Our DAP-seq, Y1H, and EMSA analyses demonstrated that ZmbHLH81 directly binds to the promoter region sequences of both *ZmSnRK2.9* and *ZmNAC20* to activate their transcription. This simultaneous transactivation of a kinase and a transcription factor suggests that *ZmbHLH81* regulates distinct components of the drought response. While SnRK2 kinases are typically involved in the physiological execution of stomatal closure, transcription factors like *ZmNAC20* operate at the transcriptional level to further promote stomatal closure and regulate broader stress-responsive gene expression [42]. This indicates that *ZmbHLH81* functions upstream of both the rapid kinase-mediated physiological responses and the NAC-mediated transcriptional regulation. This provides a molecular basis for the observed stomatal phenotypes, although the exact interactions between these components and the broader ABA signaling network remain to be further explored.

### 3.3. ZmbHLH81 Modulates Antioxidant Capacity by Directly Activating ZmHDZ4

Drought stress often induces the accumulation of ROS, and plants rely on antioxidant enzymes to alleviate oxidative damage [43]. In this study, the *ZmbHLH81* mutants exhibited significantly lower activities of SOD and POD than the WT following drought treatment. Consequently, the mutant lines accumulated significantly higher levels of MDA, reflecting more severe membrane lipid peroxidation and oxidative injury. Consistent with these physiological observations, our transcriptomic data showed that genes downregulated in the mutant were enriched in GO categories such as “oxidoreductase activity” and “heme binding”. Given that plant class III PODs are typical heme-dependent enzymes requiring heme as an essential catalytic cofactor to scavenge ROS [44], the suppression of these genes perfectly explains the reduced POD activity observed in the mutant. These results indicate that *ZmbHLH81* is involved in the antioxidant response during water deficit.

To identify the responsible downstream targets, our molecular assessments revealed that ZmbHLH81 directly binds to the promoter of the transcription factor gene *ZmHDZ4* and activates its transcription. A recent functional study demonstrated that *ZmHDZ4* improves drought tolerance in maize seedlings, and its overexpression significantly enhances SOD and POD activities to reduce oxidative damage [45,46]. The biological function of *ZmHDZ4* aligns with our physiological and transcriptomic results. By directly activating *ZmHDZ4*, ZmbHLH81 functions upstream to modulate the ROS-scavenging system. This transcriptional cascade provides a molecular explanation for how *ZmbHLH81* maintains cellular antioxidant capacity and protects maize seedlings under drought stress.

### 3.4. Limitations and Perspectives

While the current study highlights the positive role of *ZmbHLH81* in drought responses, the phenotypic evaluations were primarily restricted to the seedling stage. Considering that water deficit severely impairs crop productivity during the reproductive and grain-filling phases [7,47], the actual performance of *ZmbHLH81* requires validation under field conditions throughout the entire developmental cycle. Furthermore, the overexpression assays were conducted using the heterologous *Arabidopsis* system. Generating homologous maize overexpression lines will be essential to fully elucidate its agronomic value and to assess any potential effects on final grain yield.

Beyond these agronomic evaluations, the upstream regulatory networks governing *ZmbHLH81* remain to be characterized. Identifying the specific stress sensors or upstream factors that perceive water deficit signals to activate *ZmbHLH81* expression would help to further complete our biological understanding of this molecular cascade. Despite these unresolved upstream mechanisms, the ability of *ZmbHLH81* to simultaneously coordinate stomatal movement and ROS scavenging makes it a valuable target for crop improvement. Therefore, an essential next step involves exploring the natural variations in the *ZmbHLH81* locus across diverse maize germplasm [48]. Identifying favorable alleles associated with natural environmental adaptation could provide direct and reliable genetic resources for marker-assisted selection in future maize breeding programs.

## 4. Materials and Methods

### 4.1. Plant Materials, Growth Conditions, and Stress Treatments

The maize (*Zea mays* L.) inbred lines B73 and B104, and *Arabidopsis thaliana* (Col-0) were used in this study. *Arabidopsis* seeds were surface-sterilized, stratified at 4 °C for 2–3 days, and sown on 1/2 MS solid medium. Seven-day-old seedlings were transferred to soil (soil:vermiculite = 3:1) and grown in a controlled growth chamber (22 °C, 16 h light/8 h dark cycle, 60–70% relative humidity). Maize seeds were sown in a soil mixture (soil:vermiculite = 3:1) and grown in a greenhouse (28 °C, 16 h light/8 h dark cycle, 60% relative humidity).

For tissue-specific expression analysis, samples including roots, stems, and leaves at the V3 and V7 stages, tassels at the V18 stage, silks and ears at the R1 stage, and whole seeds at 6, 10, and 20 days after pollination (DAP) were collected from WT B73 plants grown under normal conditions. For stress treatments, B73 seedlings at the three-leaf stage were treated with either 10% PEG6000 to drive osmotic stress or 100 µM ABA. Leaves were harvested at 0, 1, 3, 6, and 12 h post-treatment, immediately frozen in liquid nitrogen, and stored at −80 °C for RNA extraction.

### 4.2. Vector Construction and Generation of Transgenic Plants

To generate *ZmbHLH81*-overexpressing *Arabidopsis* lines (OE), the full-length coding sequence (CDS) of *ZmbHLH81* (Zm00001eb036040) was amplified from B73 leaf cDNA and cloned into the *Kpn* I and *Bam* H I sites of the pCAMBIA1301 vector under the control of the CaMV35S promoter using Gibson assembly. The recombinant construct (p35S::*ZmbHLH81*) and the empty vector (EV) were introduced into *Agrobacterium tumefaciens* strain GV3101 and transformed into Col-0 using the floral dip method. Positive transformants were screened on 1/2 MS medium containing 25 µg/mL hygromycin B. T3 homozygous lines (OE-1, OE-2, and OE-3) were used for subsequent assays. To generate maize CRISPR mutants, two single guide RNAs (sgRNAs) targeting the first and fifth exons of *ZmbHLH81* were designed using CRISPR-P 2.0 (http://crispr.hzau.edu.cn/cgi-bin/CRISPR2/CRISPR, (accessed on 4 May 2021)). The sgRNA cassettes driven by the *OsU3t* and *TaU3p* promoters were assembled into the pBUE411 vector [49]. All primers used for vector construction and Y1H assays are detailed in Supplementary Table S2. The resulting construct was transformed into *Agrobacterium tumefaciens* strain EHA105 and subsequently introduced into immature embryos of the B104 inbred line via *Agrobacterium*-mediated transformation. Transformant lines were verified by PCR and Sanger sequencing. T2 homozygous mutant lines (CR-6, CR-9, and CR-11) carrying frameshift mutations were selected for further analysis.

### 4.3. Subcellular Localization

To determine the subcellular localization of ZmbHLH81, its CDS without the stop codon was fused to the N-terminus of the green GFP in the pGFP expression vector driven by a ubiquitin promoter. Maize protoplasts were isolated from the leaves of 10-day-old etiolated B73 seedlings. The pGFP-ZmbHLH81 fusion construct or the empty pGFP vector was transfected into the protoplasts using the PEG-mediated transformation method described previously [50]. After 12–16 h of incubation in the dark at 25 °C, the GFP fluorescence signals were observed and imaged using a laser scanning confocal microscope (Nikon, Tokyo, Japan).

### 4.4. Drought Tolerance Evaluation

For the drought tolerance assay in *Arabidopsis*, 20-day-old EV and OE lines were subjected to drought stress by withholding water. When severe wilting was observed in approximately 50% of the EV plants, all plants were re-watered. Survival rates were recorded 7 days after re-watering. Plants that successfully regained turgor and produced new green leaves were scored as surviving. The survival rate was calculated from three independent biological replicates (plates), with 16 seedlings per replicate.

For the drought tolerance assay in maize, seeds were pre-germinated, and 10 healthy, uniform seedlings per genotype (WT and CR) were selected and co-planted side-by-side in the same pots. At the three-leaf stage, the seedlings were subjected to drought stress by completely withholding water. Upon the appearance of severe wilting and leaf rolling phenotypes, the plants were re-watered. After 5 days of recovery, the mean survival rates were calculated from 3 to 5 independent biological replicates (pots).

### 4.5. Measurements of Water Loss, Stomatal Aperture, and Biochemical Indicators

To determine the water loss rate, detached leaves from WT and CR seedlings at the three-leaf stage were placed on paper at room temperature (25 °C). The fresh weights of the leaves were measured at 0, 2, 4, 6, 8, 10, and 12 h. The water loss rate was calculated according to the following formula: Water loss rate (%) = [(Initial fresh weight − Fresh weight at each time point)/Initial fresh weight] × 100.

For stomatal aperture analysis, leaf segments were incubated in a stomatal opening buffer (10 mM KCl, 50 µM CaCl_2_, 10 mM MES-Tris, pH 5.6) for 3 h in the dark to induce complete stomatal opening. The leaves were then exposed to light (150 μmol m^−2^ s^−1^) for dehydration. Stomatal apertures were observed at 0, 10, 20, and 60 min by making epidermal impressions with transparent nail polish. Images were captured using a Nikon Eclipse E100 optical microscope (Nikon, Tokyo, Japan), and the stomatal apertures were measured.

For physiological and biochemical assays, fresh leaf samples (0.1 g) from normal and drought-treated maize seedlings were homogenized in pre-cooled extraction buffers. The activities of SOD and POD, as well as the content of MDA, were spectrophotometrically determined using specific commercial assay kits (BC0175, BC0095 and BC0025, Solarbio, Beijing, China) following the manufacturer’s instructions.

### 4.6. RNA Extraction and qRT-PCR

Total RNA was extracted from the frozen tissues using TRIzol reagent (Invitrogen, Carlsbad, CA, USA). During sampling, tissues were thoroughly ground in liquid nitrogen, and approximately 100 mg of the fine powder was utilized for RNA isolation. Genomic DNA contamination was removed by DNase I treatment. For quantitative real-time PCR (qRT-PCR), 1 µg of total RNA was reverse-transcribed into cDNA using the TransScript^®^ One-Step gDNA Removal and cDNA Synthesis SuperMix (TransGen Biotech, Beijing, China). qRT-PCR was performed on a StepOnePlus™ Real-Time PCR System (Applied Biosystems, Foster City, CA, USA) using the PowerUp™ SYBR™ Green Master Mix (A25741; Applied Biosystems, USA), with *ZmEF1α* serving as the internal reference. The specific primers used for qRT-PCR analysis are listed in Supplementary Table S2. Relative gene expression was calculated using the 2^−ΔΔCt^ method [51].

### 4.7. RNA-Seq and Transcriptomic Analysis

Leaf samples were collected from three-leaf stage seedlings of the WT and the *ZmbHLH81* mutant line (CR-6) under drought stress, with three biological replicates. Total RNA extraction was performed as described above. Library construction and dnbSeq sequencing were performed by BGI (Shenzhen, China). High-quality clean reads were mapped to the maize B73 reference genome (RefGen_v5) using HISAT2 (version 2.2.1) [52]. DEGs were identified using the DESeq2 R package with the criteria of a fold change >1.5 (or <0.67) and FDR < 0.05 [53]. GO enrichment analysis of the DEGs was conducted using the agriGO v2.0 toolkit [54].

### 4.8. DAP-Seq

The genome-wide binding sites of ZmbHLH81 were profiled using the DAP-seq methodology [55]. The Halo-ZmbHLH81 fusion protein was heterologously expressed in *E. coli* BL21 (DE3) using the pFN19A vector and purified with HaloTag^®^ magnetic beads. Simultaneously, a genomic DNA library was constructed by fragmenting B73 seedling DNA into 200–300 bp segments, which were then end-repaired, A-tailed, and ligated to standard Illumina adapters. The purified protein was allowed to interact with the DNA library in vitro, and the specific protein-DNA complexes were directly pulled down using HaloTag^®^ magnetic beads. After stringent washing procedures, the enriched fragments were recovered, amplified, and sequenced via the Illumina NovaSeq platform. For data analysis, the filtered reads were mapped to the maize B73 genome (RefGen_v5) utilizing BWA. Finally, MACS2 was applied for peak calling (*p* < 10^−5^), and the enriched binding motifs were characterized using MEME-ChIP (version 5.5.7) [56].

### 4.9. Yeast Transactivation and Y1H Assays

To assess the transcriptional activity of ZmbHLH81, its CDS was inserted into the pGBKT7 vector to generate the BD-ZmbHLH81 construct. The recombinant plasmid and the empty BD vector were transformed into the yeast strain AH109. Transformants were initially grown on SD/-Trp medium and subsequently spotted onto SD/-Trp/-Ade and SD/-Trp/-Ade/-His media supplemented with X-α-Gal to evaluate the transactivation activity based on cell growth and blue color development.

Y1H assays were performed to verify the binding of ZmbHLH81 to target promoters. The *ZmbHLH81* CDS was cloned into the pJG4-5 vector (AD-ZmbHLH81). Promoter fragments containing the G-box motifs were cloned into the pLacZi2μ reporter vector. The effector and reporter plasmids were co-transformed into the yeast strain EGY48. Positive transformants screened on SD/-Trp/-Ura medium were evaluated for β-galactosidase activity on media containing X-gal.

### 4.10. EMSA

EMSAs were performed to confirm the in vitro binding specificity. To obtain the purified GST-ZmbHLH81 fusion protein, the full-length coding sequence of *ZmbHLH81* was cloned into the pGEX-4T-1 prokaryotic expression vector. The recombinant plasmid was transformed into *E. coli* BL21 (DE3). Protein expression was induced by the addition of 0.5 mM IPTG, followed by incubation at 25 °C for 5 h. The bacterial cells were harvested and thoroughly lysed using high-pressure homogenization. Subsequently, the recombinant GST-ZmbHLH81 protein was purified from the cell lysate using Glutathione Sepharose™ 4 Fast Flow resin (17-5132-01; Cytiva, Marlborough, MA, USA) according to the manufacturer’s instructions. Biotin-labeled DNA probes containing the G-box motifs were synthesized. The EMSA reactions were carried out using the LightShift™ Chemiluminescent EMSA Kit (Thermo Scientific, Waltham, MA, USA). For competition assays, 10-fold and 100-fold excesses of unlabeled competitor probes were added to the binding reactions before the addition of the labeled probes. The protein-DNA complexes were separated on a 6% native polyacrylamide gel and detected using a chemiluminescence imaging system.

## Data Availability

Data are contained within the article and Supplementary Materials.

## Supplementary Materials

The following supporting information can be downloaded at: https://www.mdpi.com/article/10.3390/ijms27073293/s1.

## Abbreviations

The following abbreviations are used in this manuscript:

ABA	abscisic acid
bHLH	basic helix-loop-helix
DAP-seq	DNA affinity purification sequencing
EMSA	electrophoretic mobility shift assay
FDR	false discovery rate
GFP	green fluorescent protein
GO	Gene Ontology
GST	glutathione S-transferase
MDA	malondialdehyde
POD	peroxidase
ROS	reactive oxygen species
sgRNA	single guide RNA
SOD	superoxide dismutase
TSS	transcription start site
