# Supplementary material for: ZmbHLH81 Enhances Maize Drought Tolerance via Direct Transcriptional Activation of ABA Signaling and ROS Scavenging Genes
Source: Int J Mol Sci. 2026 Apr 5;27(7):3293. doi: 10.3390/ijms27073293 (PMC13074027; doi:10.3390/ijms27073293)
Supplement: Supplementary file 1 [file ijms-27-03293-s001.zip › Supplementary_Figure_S1.pdf]

```
CR-6        MKEGRPGAGDYIASLLSSSPRLDFGGEEEDCLDRFCGDPGFAARAARLSSF*RAALRRH 59
CR-11       MKEGRPGAGDYIASLLSSSPRLDFGGEEEDCLDRFCGDPGFAARAARLS---RAALRRH 57
WT          MKEGRPGAGDYIASLLSSSPRLDFGGEEEDCLDRFCGDPGFAARAARLSSFSGQRFAVT 60
CR-9        MKEGRPGAGDYIASLLSSSPRLDFGGEEEDCLDRFCGDPGFAARAARLSSLAGSASPSP 60
            *************************************************

CR-6        RGPLRAAPAAARRERRRRVRRVPGGVVGVGPGVGDEGRQCQEAEGARGGGGQGQRQGAIC 119
CR-11       RGPLRAAPAAARRERRRRVRRVPGGVVGVGPGVGDEGRQCQEAEGARGGGGQGQRQGAIC 117
WT          AGLFGLPPPLPAA---------SGGGEFAGSR--E-ASSV-------------------- 88
CR-9        RASSGCPRRCPPR---------AAAASSPGPG--R-RRRCRTRR---------------- 92
             .                     ..    *

CR-6        AGTGRGAEGSGRQEVLQGGGRRGGGGEPREAAQAGAGRQRQLRRGRRRAEPEAAAAGEGK 179
CR-11       AGTGRGAEGSGRQEVLQGGGRRGGGGEPREAAQAGAGRQRQLRRGRRRAEPEAAAAGEGK 177
WT          ---------SDPASAMKDA-NAKKRKAPAAAAAKGKGREPSA--QAQAGEPKGPDA--KR 134
CR-9        ---------RR*RTPMPRSGRRPRRRRPRAKAGSHLRRHRQG--SRRVRTPRGAAR--R- 137
                           :  . .      *   *     *. .     :   *..

CR-6        ECQAG---GASQGLRPCPGQERAGYRQP------------------QPRREGEKGED--- 215
CR-11       ECQAG---GASQGLRPCPGQERAGYRQP------------------QPRREGEKGED--- 213
WT          CCKAEGGEGEEGSPVKLPKPEQAGSDSSVEDGGAQ--------NQKPPPP--VKGKN--A 182
CR-9        ----REARGRRGAP*SCPSRSRPAATAPSRTAARRTRSRRRR*RERMPSRWSLPGTTSMS 191
                    *   .    *  .: .                       *      *

CR-6        QPEDEGPSGPGARMQQGDRQGADARRDHKLRAVAATAGRVPLHEARNRQPTRT*ATCPRS 274
CR-11       QPEDEGPSGPGARMQQGDRQGADARRDHKLRAVAATAGRVPLHEARNRQPTPEQPAHAPT 273
WT          KPVE--PPRDYV-HVRA--RRGQATDSHSLAERVR-RERIS---QRMKVLQDLVPGCNKV 233
CR-9        GPGEGRLPTATA-SQRG*EGRGSARG*RSFRTWCQDATR*S---ARR*CSTRS*TTCSRC 242
             * :       .   :.    ..*   :.:        *      *

CR-6        YKKICSRPAALRRARSSRWRAAARGSRSAGKGTCSSPSSRTAWRTRAAA*TRWTWLCPRL 333
CR-11       KRYVP----GLRRFGELGLL---AGELQP-----GVPVRRA--------RGRVPVLRPER 313
WT          IGKAL----MLDE--IINYVQ----SLQR--QVEFLSMKL---------ATVNPLDLSNL 272
CR-9        NGRS---------------------------SSSP*SS---------QPSTHWDLSNL 263
                                                                      .

CR-6        PAG----------SSVSRTERPAQTCSKGTTGRRRR--RTC-------RACSTS------ 368
CR-11       PG-----------EPVRRPE-PA--GPGSVPGYRRA--VRFPGRNGRHKPAAKE------ 351
WT          PTLLQKDMFQACGASAS-SVFSLESCSPGFPFGGQGDVFQSFVPNGLENPCGGLNPLDLA 331
CR-9        PTLLQKDMFQACGASAS-SVFSLESCSPGFPFGGQGDVFQSFVPNGLENPCGGLNPLDLA 322
            *              .            .     :             . ..

CR-6        ---TTTG----RARSTGPQLQH-------KASMVSFNHKRA---T*RWSS-------S-- 401
CR-11       ---LLGG----GGGGPAERVPHRRQRAEPGARGLSFS---T---KLPWSA-------STT 391
WT          LSQATGGQFGFQDGTAGTNLQQRNYWEEEEEDLQSVFHIDDNGQSQEHGASASAQSFHGQ 391
CR-9        LSQATGGQFGFQDGTAGTNLQQRNYWEEEEEDLQSVFHIDDNGQSQEHGASASAQSFHGQ 382
                  *         . .: :            *.        .   .:

CR-6        --------------       401
CR-11       RGPHEDGVLL----       401
WT          LQPQEGHMKMEFF*       404
CR-9        LQPQEGHMKMEFF*       395
```

Figure S1 Protein sequence alignment of the WT and the three *ZmbHLH81* mutant lines. The CRISPR/Cas9-mediated frameshift mutations lead to premature translation termination in the CR-6 and CR-9 lines, with black boxes indicating the positions of the premature stop codons. In contrast, the mutation in the CR-11 results in a completely altered amino acid sequence downstream of the first target site. Both the premature truncation and the sequence alteration are strongly predicted to severely disrupt the function of the ZmbHLH81 protein.
